# Pharmacological Inhibition of Toll-Like Receptor-4 Signaling by TAK242 Prevents and Induces Regression of Experimental Organ Fibrosis

**DOI:** 10.3389/fimmu.2018.02434

**Published:** 2018-10-23

**Authors:** Swati Bhattacharyya, Wenxia Wang, Zenshiro Tamaki, Bo Shi, Anjana Yeldandi, Yasuhiro Tsukimi, Masashi Yamasaki, John Varga

**Affiliations:** ^1^Northwestern Scleroderma Program, Feinberg School of Medicine, Chicago, IL, United States; ^2^Takeda Pharmaceutical Company Limited, Tokyo, Japan

**Keywords:** systemic sclerosis, SSc, fibrosis, IL-6, toll-like receptor, TLR, TAK242, resatorvid

## Abstract

Systemic sclerosis (SSc) is a poorly understood heterogeneous condition with progressive multi-organ fibrosis. Recent genetic and genomic evidence suggest a pathogenic role for dysregulated innate immunity and toll-like receptor (TLR) activity in SSc. Levels of both TLR4, as well as certain endogenous TLR ligands, are elevated in skin and lung tissues from patients with SSc and correlate with clinical disease parameters. Conversely, genetic targeting of TLR4 or its endogenous “damage-associated” ligands ameliorates progressive tissue fibrosis. Targeting TLR4 signaling therefore represents a pharmacological strategy to prevent intractable fibrosis. We examined the effect of TAK242, a small molecule TLR4 inhibitor, in preclinical fibrosis models and in SSc fibroblasts. TAK242 treatment prevented, promoted regression of, bleomycin-induced dermal and pulmonary fibrosis, and reduced the expression of several pro-fibrotic mediators. Furthermore, TAK242 ameliorated peritoneal fibrosis and reduced spontaneous hypodermal thickness in TSK/+ mice. Importantly, TAK242 abrogated collagen synthesis and myofibroblasts differentiation in explanted constitutively active SSc fibroblast. Altogether, these findings identify TAK242 as an anti-fibrotic agent in preclinical models of organ fibrosis. TAK242 might potentially represent a novel strategy for the treatment of SSc and other fibrotic diseases.

## Introduction

A unique feature of SSc is synchonous fibrosis in multiple organs. Despite recent advances in understanding cellular and molecular basis of fibrosis, SSc still carries significant mortality. Most SSc patients develop interstitial lung disease (ILD), which currently accounts for >30% of all SSc deaths (1). There are no therapies effective in halting or resolving fibrosis (2). While multiple intracellular signaling pathways are implicated in SSc fibrosis, the nature of their persistent deregulation in pathological inflammation and fibrosis remains poorly understood (3). We recently showed that toll-like receptor 4 (TLR4) and its endogenous ligands damage associated molecular patterns (DAMPs) such as fibronection-EDA and tenascin C are markedly elevated in SSc skin and lungs (4–6). Conversely, genetic targeting of TLR4 or its endogenous DAMPs, was shown to ameliorate tissue fibrosis in multiple preclinical models of SSc (4–6). Stimulation of fibroblasts TLR4 was associated with the induction of extracellular matrix (ECM) remodeling and tissue repair programs, as well as synergistic enhancement of TGF-beta-mediated fibrotic responses (6). Thus, in a fibrogenic milieu enriched with both TGF-beta and endogenous TLR4 ligands, fibroblasts expressing elevated TLR4 are likely to engage in uncontrolled ECM production and myofibroblast differentiation, contributing to progression of fibrosis. Disrupting persistent TLR4 signaling with TLR4 inhibitors therefore might represent a potential strategy for breaking the vicious fibrosis cycle in SSc or other fibrotic diseases (7, 8).

TAK242 is a small molecule inhibitor of TLR4 signaling that blocks the production of lipopolysaccharide-induced inflammatory mediators (9). TAK242 binds to Cys747 in the intracellular domain of TLR4, and disrupts the interaction of TLR4 with adaptor molecules, thereby inhibiting TLR4 signaling (9, 10). While TAK-242 was shown to suppress the increase in serum TNF-a, IL-1, and IL-6 in murine and porcine models of sepsis, it had no effect on survival (10, 11). In published clinical trials of sepsis, TAK-242 demonstrated non-significant reduction in patient mortality, but failed to demonstrate a beneficial effect on inflammatory cytokine levels. Moreover, treatment was associated with methemoglobinemia in some patients (12). Therefore, TAK-242 does not appear to be an optimal therapy for the treatment of sepsis, whereas its potential efficacy and safety in other diseases linked to excessive TLR4 signaling remain to be investigated. The strategy of converting the indications of existing drugs from one therapeutic area to include the treatment of other diseases, known as “drug repurposing” curtails the time required for clinical application. In view of our recent findings implicating the DAMP-TLR4 axis in driving sustained fibroblasts activation underlying fibrosis progression in SSc, we sought to investigate the effect of TAK242 in preclinical models of organ fibrosis, and in SSc fibroblasts. Our results demonstrate that selective pharmacological blockade of TLR4 signaling with TAK242 prevented and reversed organ fibrosis in multiple distinct mouse models. Moreover, TAK242 reduced constitutive collagen synthesis and myofibroblasts transdifferentiation in explanted SSc fibroblasts. These results provide a rationale for further exploring selective targeting of TLR4 signaling as a potential therapeutic strategy in patients with SSc.

## Materials and methods

### Cell culture and reagents

Primary cultures of human fibroblasts were established by explantation from patients with SSc, healthy, or mouse skin (6). Biopsies were performed with written informed consent and in accordance with protocols approved by the Institutional Review Board for Human Studies at Northwestern University. Mouse fibroblast cultures were established from skin biopsy specimens of 8-week-old female TLR4-mutant C3H/HeJ mice and WT control C3H/HeOuJ mice (Jackson Laboratories, Sacramento, CA) and studied in parallel. Low-passage fibroblasts were grown in monolayers in plastic dishes, and studied at early confluence. Cultures were maintained in Dulbecco's Modified Eagle's medium (DMEM) supplemented with 10% fetal bovine serum (FBS) (Gibco BRL, Grand Island, NY), 1% vitamin solutions, and 2 mM L-glutamine. All other tissue culture reagents were from Lonza (Basel, Switzerland). TAK242 was synthesized as previously reported by TAKEDA (Osaka, Japan) (10). For experiments using SSc fibroblasts, cultures were placed in low serum media containing 0.1% FBS with or without the TLR4 inhibitor TAK242. For other experiments, cultures were placed in serum-free media containing 0.1% bovine serum albumin (BSA) with or without indicated concentrations of TAK242. The inhibitor was added 60 min prior to endotoxin-free Fn^EDA^ (1 μg/ml) isolated and purified from embryonic IMR90 fibroblasts (4).

### Cytotoxicity assays

Cell death or cytotoxicity was evaluated by quantification of plasma membrane damage using LDH Cytotoxicity Assay Kit II (Colorimetric Assay Kits, Biovision, Milpitas, CA).

### Experimental models of fibrosis

Animal experiments were performed according to institutionally approved protocols and in compliance with guidelines of the Northwestern University Animal Care and Use Committee. A series of complementary fibrosis models were employed to evaluate pharmacological TLR4 blockade *in vivo*. First, 8-week-old female C57BL/6J (The Jackson Laboratory) mice received subcutaneous (s.c.) injections of bleomycin (10 mg/kg/day) or PBS daily for 10 days (5 days/week), along with TAK242 (10 or 30 mg/kg) by daily intraperitoneal (i.p.) injections starting concurrently with bleomycin, and sacrificed on day 7 or 22. Another group of mice received TAK242 injections started at day 15, and continued until sacrifice at day 28. A third group of mice received PBS, and a fourth received bleomycin alone. In a complementary non-inflammatory fibrosis model, 6 week-old Tsk1/+ mice (C57BL/6 background, The Jackson Laboratory) received TAK242 (30 mg/kg) injections i.p. daily till sacrifice at 12 weeks.

Tissue collagen content was determined by hydroxyproline assays using Colorimetric Assay Kits (Biovision, Milpitas, CA) (5). Lung fibrosis was quantitated in histological lung sections using the modified Ashcroft score determined from 5 h.p.f. per mice (Hubner score) (13).

Peritoneal fibrosis was induced by i.p. injections of 0.1% chlorhexidine gluconate (CG) (Wako Pure Chemical Industries, Osaka, Japan) dissolved in 15% ethanol/PBS and injected every other day. C57BL/6 male mice (~25 g) received CG alone, or together with TAK242 (PO, BID; 30 or 100 mg/kg) or vehicle starting concurrently with CG injection for up to 21 day. At day 22, mice were sacrificed, and peritoneal tissues were carefully dissected. To avoid damage to the peritoneum, injections were made at the caudal part of the peritoneum, while the rostral portion of the parietal membrane was taken for analysis.

### Isolation and analysis of RNA

At the end of the experiments, total RNA was isolated and reverse-transcribed to cDNA using Supermix (cDNA Synthesis Supermix; Quanta Biosciences, Gaithersburg, MD) as described (6). Amplification products (50 ng) were amplified using SYBR Green PCR Master Mix (Applied Biosytems, Foster City, CA) on an Applied Biosystems 7500 Prism Sequence Detection System. Data were normalized to GAPDH RNA, and -fold change in samples was calculated (5).

### Transient transfection assays

The reporter constructs 772COL1A2-luc, harboring the 2772/58-bp fragment of the human proα1(I) collagen was used in transient transfection assays. Subconfluent cultures of wt and TLR4 mutant mouse skin fibroblasts in serum-free media were transfected using Superfect reagent (Qiagen, Valencia, CA). After 72 h of incubation with Fn^EDA^ (10 ug/mL) in absence or presence of TAK242 (3 uM), cultures were harvested and whole cell lysates were assayed for their luciferase activities. In each experiment, fibroblasts were cotransfected with Renilla luciferase pRL-TK plasmids (Promega, Madison, WI) as a control for transfection efficiency.

### Immunofluorescence confocal microscopy

To assess TAK242 modulation of fibroblast responses in SSc or normal skin fibroblasts were treated with TAK242 for 24 h. Cells were then fixed, permeabilized, and incubated with antibodies to type I collagen (Southern Biotech) and α-smooth muscle actin (αSMA, 1:500) (Sigma, St. Louis, MO) at 1:500 dilution, followed by Alexa-fluor-labeled secondary antibodies (Invitrogen). Nuclei were identified using 4,6-diamidino-2-phenylindone (DAPI). Subcellular distribution of immunofluorescence was evaluated under an immunofluorescence microscope or Zeiss UV Meta 510 confocal microscope (Carl Zeiss Inc, Jena, Germany) and quantitated using ImageJ (National Institutes of Health) (6). For tissue immunofluorescence, paraffin-embedded sections were incubated with primary rabbit antibodies against α-SMA (Sigma, 1:500), followed by incubation with Alexa Fluor–conjugated IgG secondary antibodies (Invitrogen). Mean fluorescence intensities were quantified by using ImageJ (means from four randomly selected hpf/subject). Sections were imaged using Zeiss UV Meta 510 confocal microscope (Carl Zeiss Inc, Jena, Germany) and quantitated using ImageJ (National Institutes of Health) (5).

### Statistical analysis

Data are presented as means ± S.D. Two-tailed Student's *t*-test or Mann Whitney test were used for comparisons between two groups. Differences among groups were examined for statistical significance using analysis of variance (ANOVA) followed by Sidak's correction. A *p-*value less than 0.05 denoted the presence of statistically significant difference. Data were analyzed using Graph Pad prism (Graph Pad Software version 5, Graph Pad Software Inc., CA).

## Results

**Figure 1:**
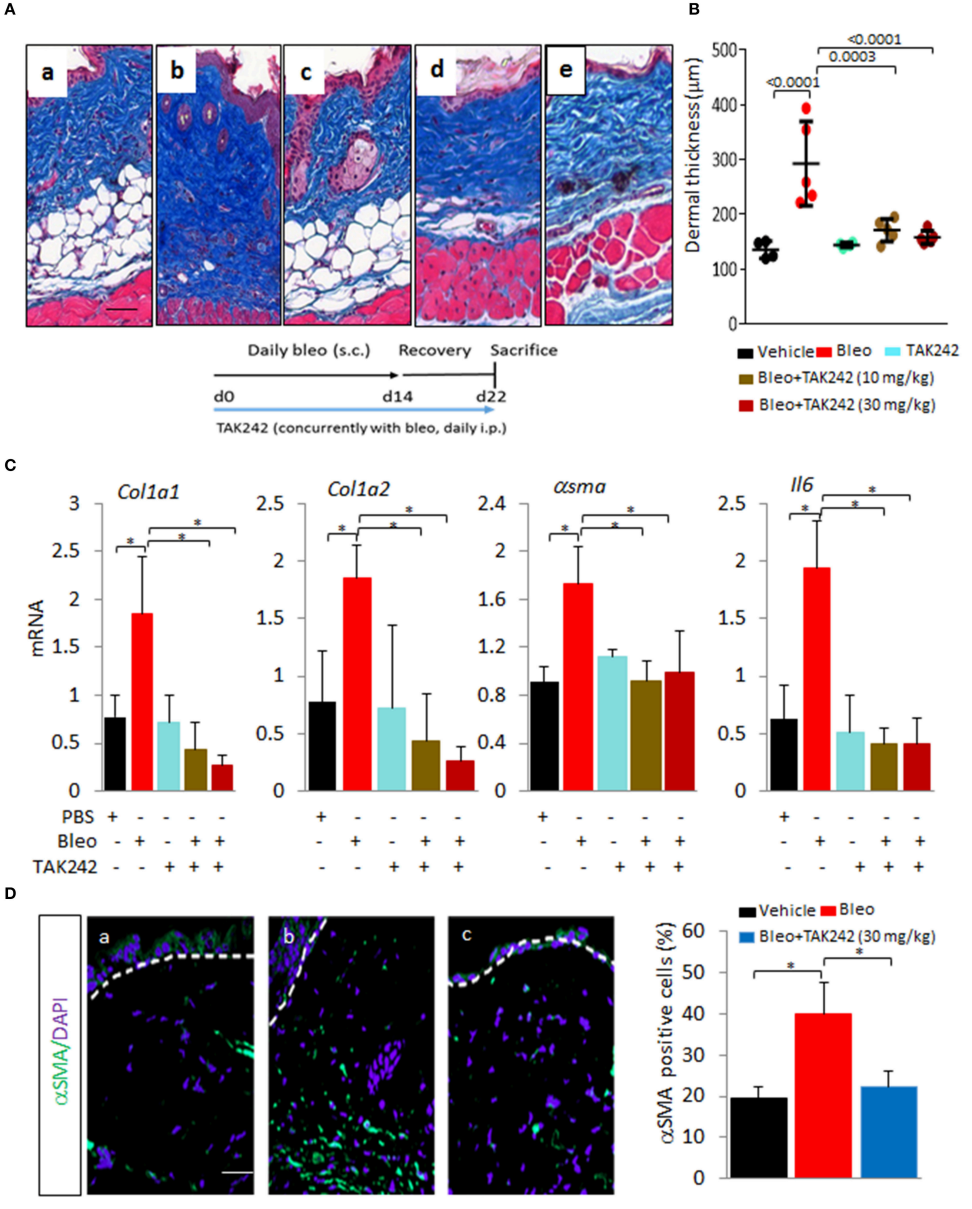
Inhibition of TLR4 signaling by TAK242 prevents skin fibrosis. C57/BL6 mice received daily s.c. injections of PBS or bleomycin alone, or together with TAK242 (10 or 30 mg/kg) or vehicle. Mice were sacrificed at day 22 and skin was harvested for analysis. **(A)** Masson's Trichome stain. (a) vehicle, (b) bleomycin for 2 weeks (5 days a week), (c) TAK242, and (d) low dose TAK242 (10 mg/kg) and bleomycin, and (e) high dose of TAK242 and bleomycin (30 mg/kg) injected concurrently for 2 weeks (5 day/week). Representative images. Bar = 25 μm. **(B)** Dermal thickness (means ± s.d. of five determinations/hpf from five mice/group). One-way analysis of variance followed by Sidak's multiple comparison test. *p < 0.05. **(C)** Real-time quantitative PCR. Results, normalized with GAPDH, are means ± s.d. of triplicate determinations from four mice/group; One-way analysis of variance followed by Sidak's multiple comparison test. *p < 0.05. **(D)** Immunofluorescence using antibodies to α-smooth muscle actin (αSMA, green) and DAPI (blue). Treatment groups as indicated inset. bleomycin alone for 2 weeks, (c) bleomycin plus TAK242 started concurrently. Representative images. Bar = 50 μm. Relative fluorescence intensities (means from four randomly selected from thee mice/group).

In light of the profibrotic activities associated with TLR4 (6, 7), we sought to evaluate the anti-fibrotic potential of TAK242 using mouse models of skin fibrosis. To induce fibrosis, we injected C57BL/6J mice with bleomycin by daily s.c. injections for 2 weeks (5 days/week). Mice were given concurrent TAK242 (10 or 30 mg/kg) or vehicle by daily i.p. injections (5 days/week) in parallel. Mice were sacrificed at day 22, and lesional skin was harvested and analyzed. Injection of TAK242 for up to 2 weeks was well tolerated by mice, and no significant weight loss or other signs of toxicity. The thickness of the dermis was markedly increased in mice injected with bleomycin compared to PBS (Figures 1A,B). Both excessive collagen deposition (left panel) and increased dermal thickness (right panel) were markedly ameliorated when TAK242 was administered concomitantly with bleomycin. Moreover, the dramatic loss of the intradermal adipose layer accompanying dermal fibrosis was substantially attenuated in mice treated with TAK242. Furthermore, up-regulated expression of multiple fibrotic marker genes (*Col1a1*,*Col1a2, asma*, and *Il6)* in lesional skin was attenuated in TAK242-treated mice (Figure 1C). Moreover, α-SMA expression in the lesional dermis notably attenuated in in TAK242-treated mice (Figure 1D).

**Figure 2:**
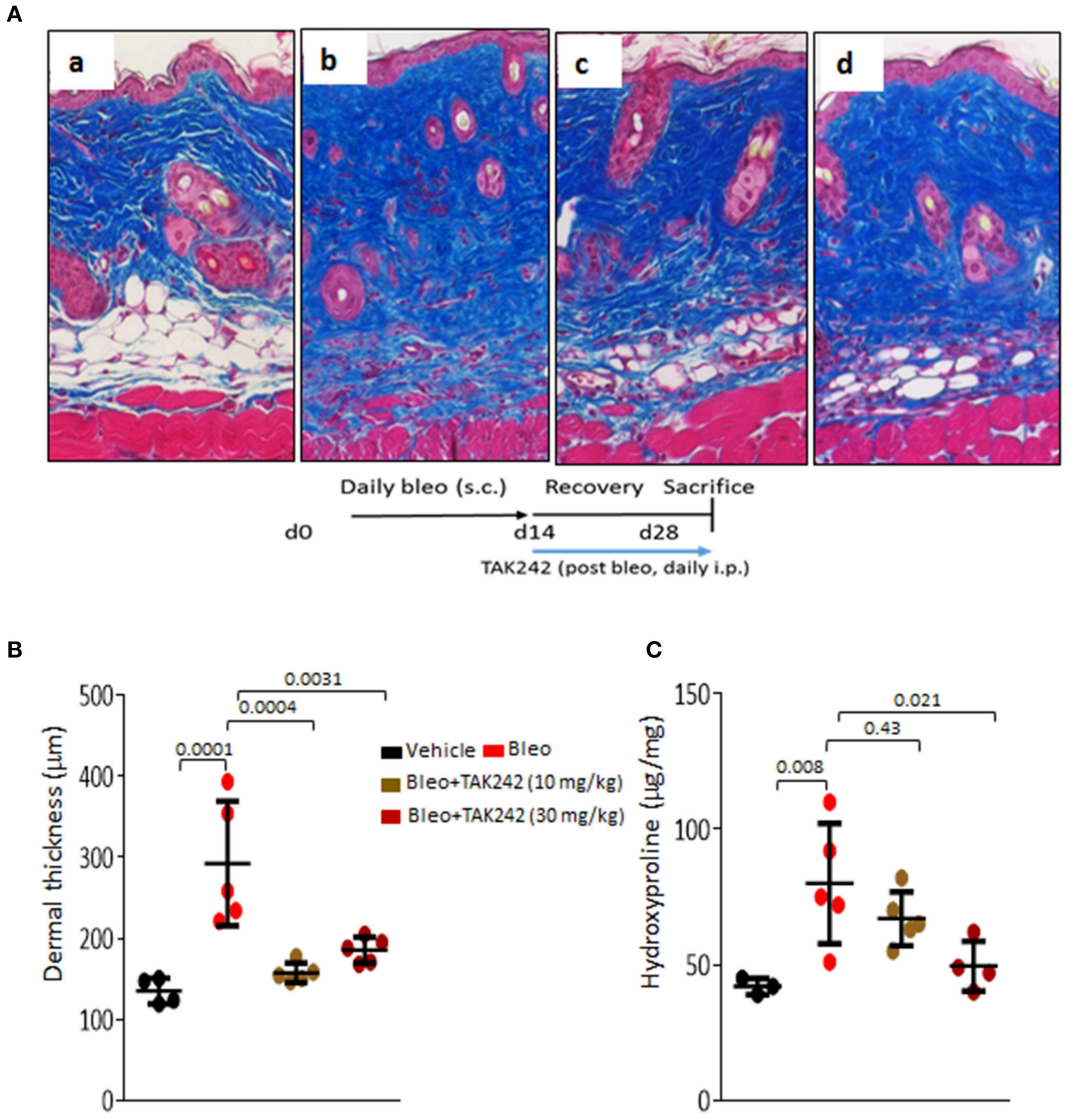
Inhibition of TLR4 signaling by TAK242 induces regression of established skin fibrosis. C57/BL6 mice received daily s.c. injections of PBS or bleomycin alone, or TAK242 (10 or 30 mg/kg) or vehicle started after 2 weeks of bleomycin injections. Mice were sacrificed at day 28 and skin was harvested for analysis. **(A)** Masson's Trichome stain. (a) vehicle, (b) bleomycin for 2 weeks (5 days a week), (c) bleomycin followed by low dose (10 mg/kg), and (d) high dose (30 mg/kg) of TAK242 post-treatment until harvest at 28 days. Representative images. Bar = 25 μm. **(B)** Dermal thickness (means ± s.d. of five determinations/hpf from five mice/group). **(C)** Skin hydroxyproline content. One-way analysis of variance followed by Sidak's multiple comparison test.

**Figure 3:**
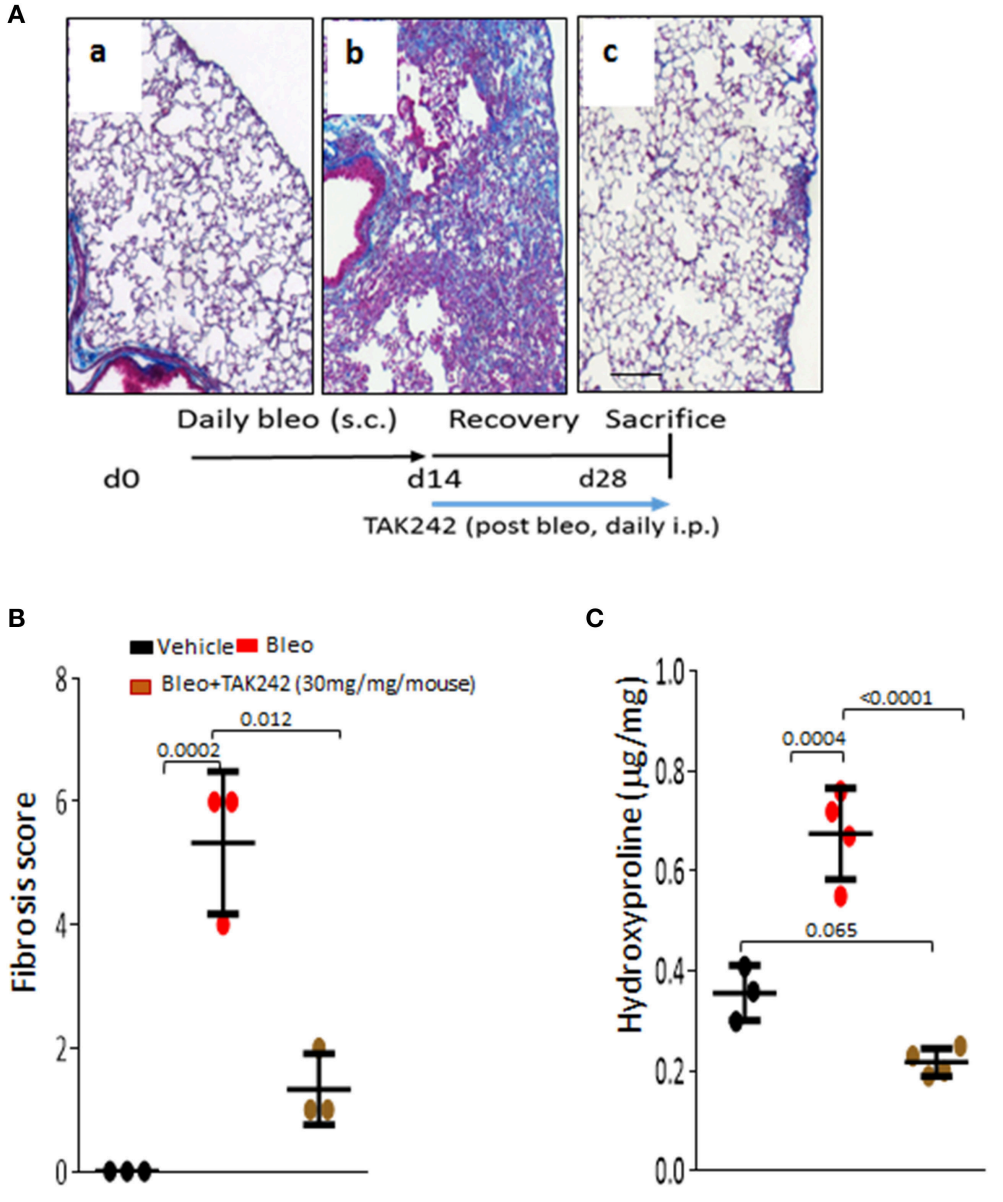
Inhibition of TLR4 signaling by TAK242 induces regression of established lung fibrosis. C57/BL6 mice received daily s.c. injections of PBS or bleomycin alone, or TAK242 (10 or 30 mg/kg) started after 2 weeks of bleomycin injections or vehicle. Mice were sacrificed at day 28 and lung was harvested. **(A)** Masson's Trichome stain. (a) vehicle, (b) bleomycin for 2 weeks (5 days a week), (c) bleomycin followed by high dose (30 mg/kg) of TAK242 post-treatment until harvest at 28 days. Representative images. Bar = 25 μm. **(B)** Fibrosis score (Hubner) (13) determined in lungs from 5 hpf per mice. Results are means±s.d. from three mice per group. One-way analysis of variance followed by Sidak's multiple comparison test. **(C)** Lung hydroxyproline content. One-way analysis of variance followed by Sidak's multiple comparison test.

Next, to determine if TLR4 inhibition might promote fibrosis regression, treatment was initiated when dermal fibrosis is already established by bleomycin treatment. The results showed that TAK242 treatment reduced the increased dermal thickness (*p* < 0.01) and hydroxyproline content, even when treatment was initiated after fibrosis has developed (Figure 2). Subsequent experiments sought to explore the role of TLR4 in lung fibrosis, a major fibrotic complication of SSc. Chonic s.c. injection of bleomycin elicited prominent lung changes, with an influx of inflammatory cells, and emergence of fibrotic foci those were primarily subpleural, along with sparse perivascular and interstitial fibrosis. Lung fibrosis was associated with substantial collagen accumulation and increase in pathological fibrosis score (Figures 3A,B). Trichome stain and hydroxyproline assays confirmed marked pulmonary accumulation of collagen (Figure 3C). Each of these parameters of bleomycin-induced pulmonary fibrosis showed substantial attenuation in TAK242-treated mice.

**Figure 4:**
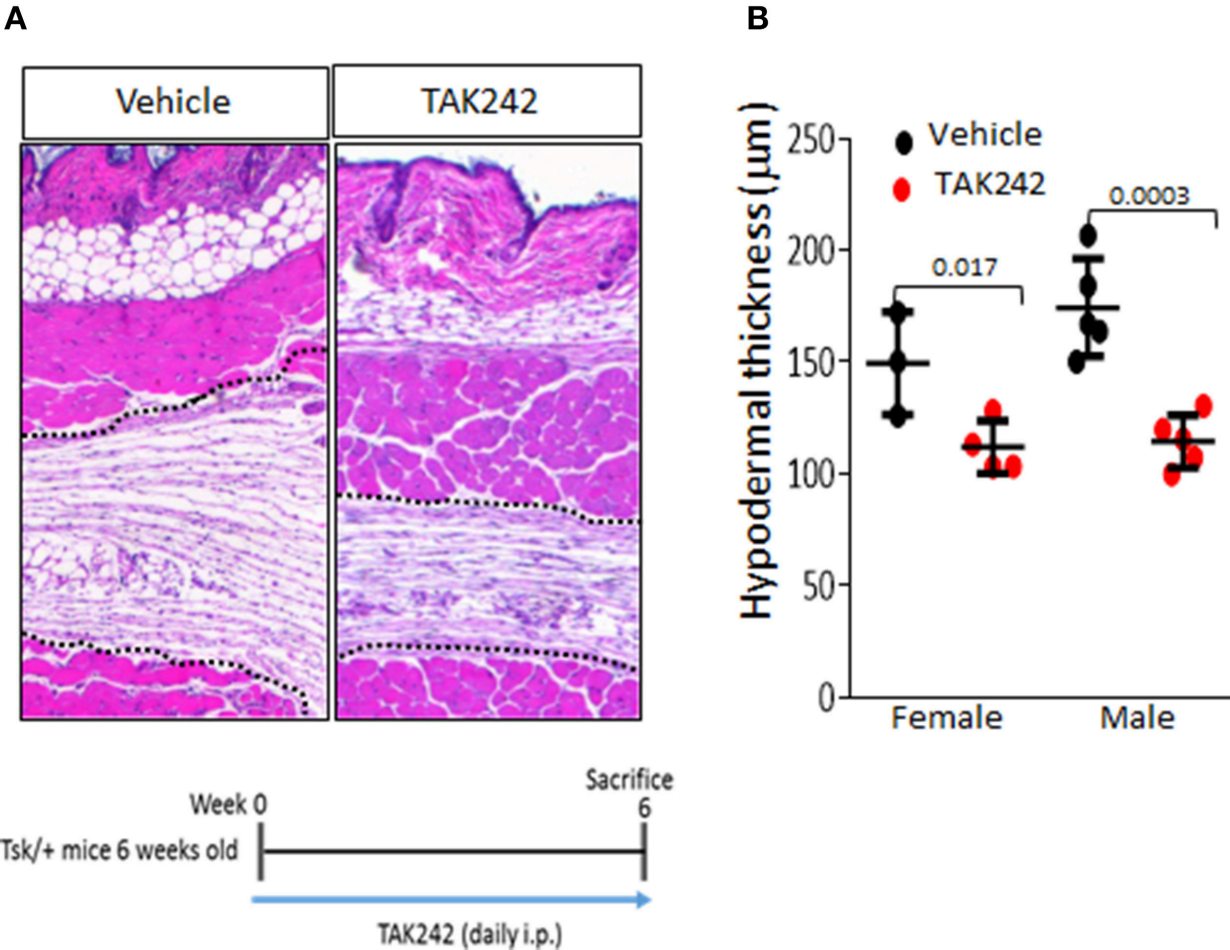
Inhibition of TLR4 signaling by TAK242 exerts antifibrotic effects in Tsk1/+ mice. Six week-old Tsk1/+ mice received TAK242 (i.p.; 30 mg/kg) daily, sacrificed at 12 weeks of age, and dorsal skin was harvested for analysis. **(A)** H and E stain (dotted line indicate hypodermis). **(B)** Hypodermal thickness (means ± s.d. of five determinations/hpf from four or five mice/group). Bar = 200 μm. Student's t-test.

In order to evaluate the effect of TLR4 inhibition in an inflammation-independent model of fibrosis, we used Tsk1/+ mice that spontaneously develop hypodermal fibrosis in the absence of inflammation (5). We therefore treated male and female TSK1/+ mice with TAK242 or vehicle in parallel. Treatment was initiated at 6 weeks of age, when skin fibrosis is already detectable, and continued for 6 weeks. When sacrificed at 12 weeks of age, TSK1/+ mice displayed a substantial increase in hypodermal thickness (5). In contrast, both male and female TSK1/+ mice that received TAK242 for 6 weeks showed significantly attenuated hypodermal thickening compared to vehicle-treated TSK1/+ mice (Figure 4). Chonic i.p. administration of TAK242 was not associated with adverse effects in either mouse strain.

### TAK242 protects mice from peritoneal fibrosis

**Figure 5:**
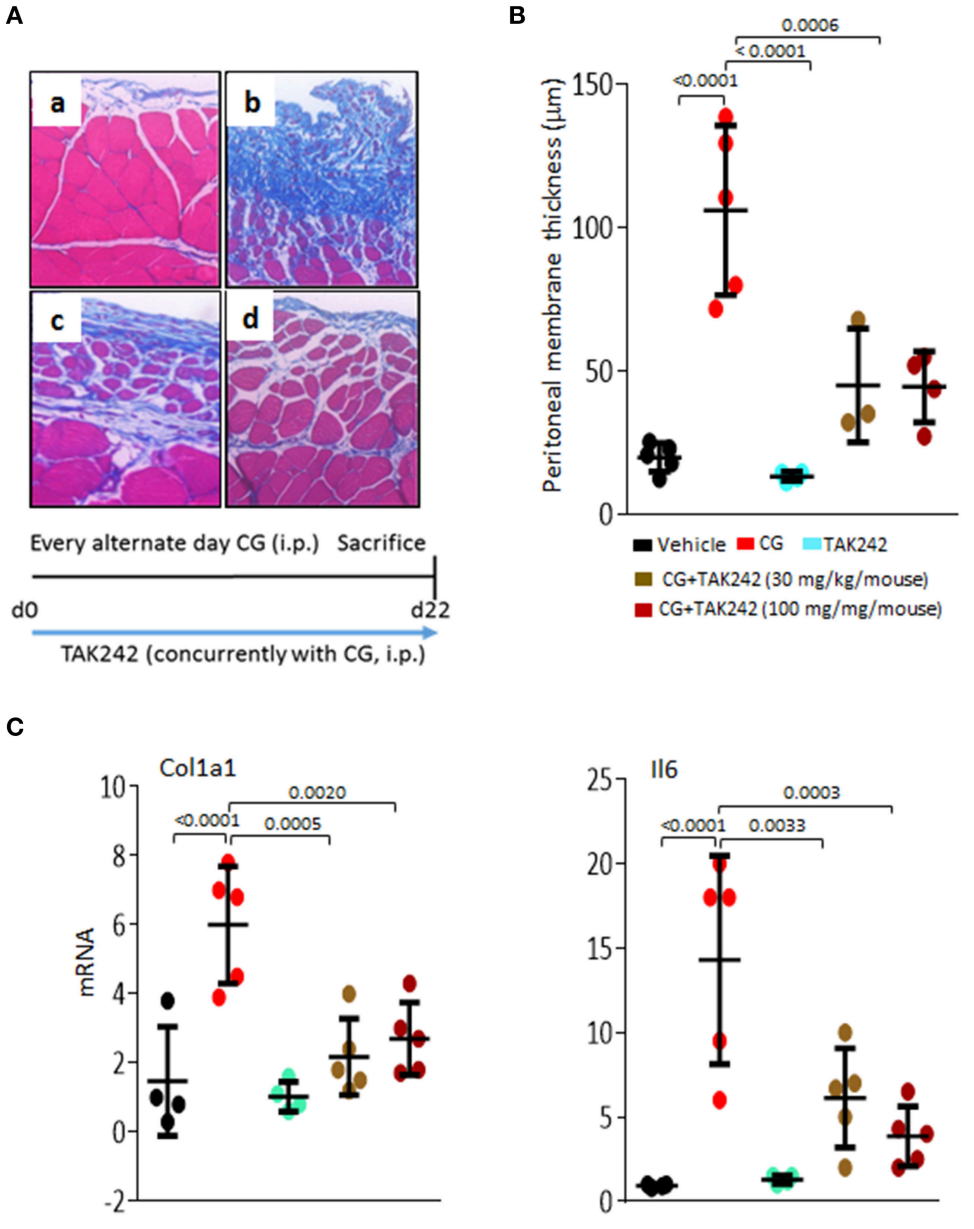
Inhibition of TLR4 signaling by TAK242 ameliorates peritoneal fibrosis. C57/BL6 mice received i.p. injections of vehicle or chlorhexidine gluconate (CG) alone (i.p.; every other day), or together with TAK242 (daily PO, BID; 10 or 30 mg/kg) started concurrently with CG injections. Mice were sacrificed at day 22, and parietal peritoneal membranes were harvested for analysis. **(A)** Left panel, Masson's trichome stain. (a) vehicle, (b) CG treatment, (c) CG plus concurrent treatment of TAK242 (low dose, 30 mg/kg; PO, BID), or (d) TAK242 (high dose, 100 mg/kg; PO, BID). Representative images. Bar = 25 μm. **(B)** Submesothelial compact zone thickness. Results are means ± s.d. of five determinations/hpf from five mice/group. One-way analysis of variance followed by Sidak's multiple comparison test. **(C)** Real-time qPCR. Results, normalized with GAPDH, represent the means ± s.d. of triplicate determinations from at least four mice/group; One-way analysis of variance followed by Sidak's multiple comparison test.

Long-term peritoneal dialysis is frequently complicated by peritoneal fibrosis, a process that can be phenocopied in mice injected with CG (14). To explore the impact of TLR4 inhibition in peritoneal fibrosis, male C57BL/6 mice were administered CG on alternate days, and treatment with daily TAK242 (PO) was initiated concurrently with CG. Mice were sacrificed at day 22 and the parietal peritoneal membranes were harvested for analysis. The results showed a nearly 5-fold increase in thickness (*p* < 0.0001) and marked accumulation of collagen, in CG-treated mice (Figure 5A). Treatment with TAK242 was highly effective in preventing fibrosis. We found >60% reduction in membrane thickness, and attenuated expression of fibrotic and inflammatory genes (*Col1a1*and *Il6*) within the fibrotic peritoneal lining (Figures 5B,C).

### TAK242 treatment mitigates the TLR4-specific profibrotic fibroblast phenotype

**Figure 6:**
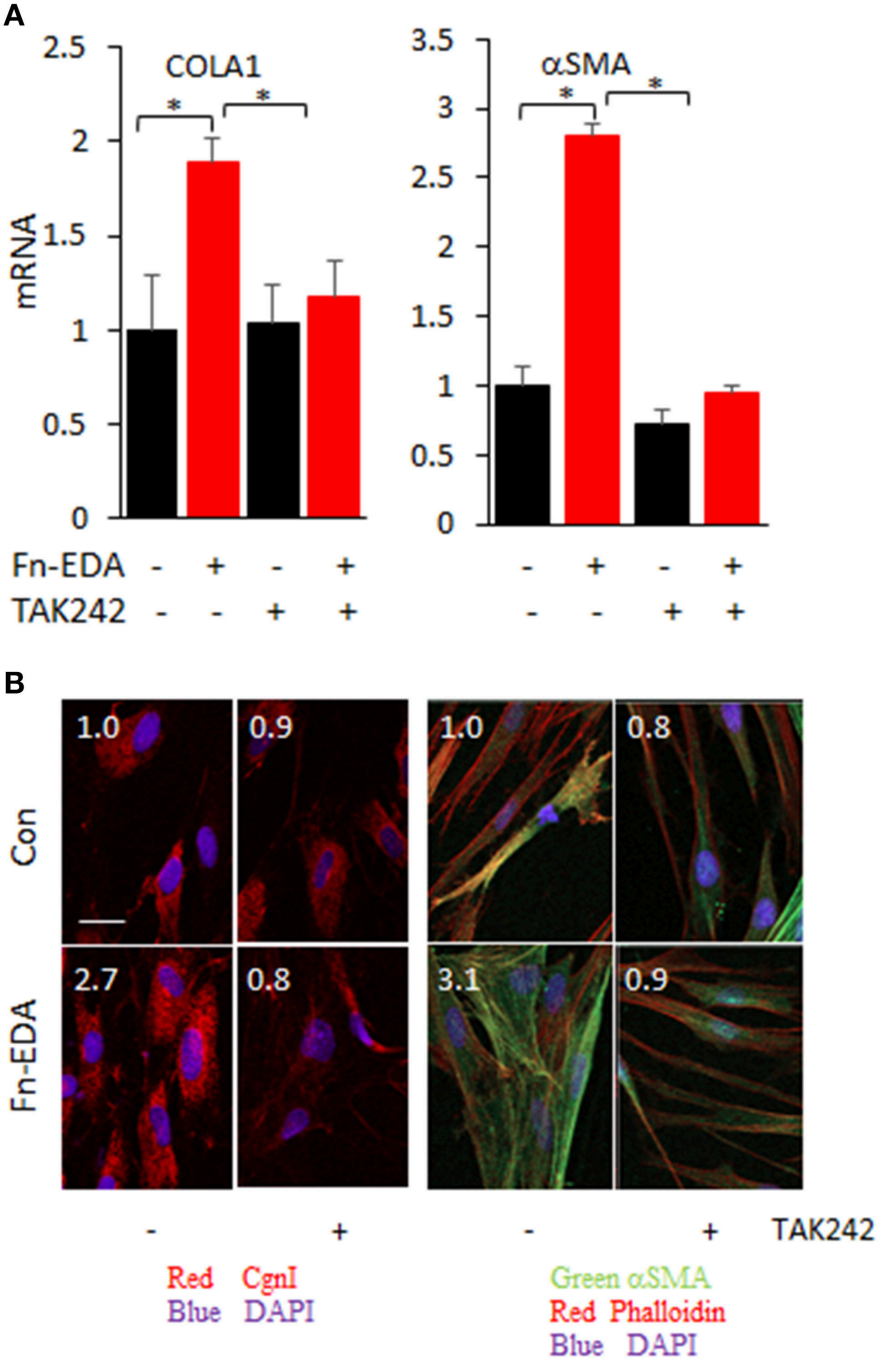
Inhibition of TLR4 signaling by TAK242 abrogated activated profibrotic fibroblast phenotype. Normal skin fibroblasts were incubated in medium with Fn^EDA^ (10 μg/ml) for 72 h in absence or presence of TAK242 (3 uM). **(A)** mRNA levels were determined by real-time qPCR. Results, normalized with GAPDH, are the means ± SD of triplicate determinations from two independent experiments (P < 0.01, one-way ANOVA followed by Sidak's multiple comparison test). **(B)** Immunofluorescence using antibodies to type I collagen (red), αSMA (green), and Phalloidin (red). Representative images. Scale bar, 50 μm. Relative fluorescence intensities (inset, means from four randomly selected field/well). *p < 0.05.

In preliminary experiments with normal fibroblasts, TAK242 showed no cytotoxicity in concentrations up to 10 μM (Supplementary Figure 1A). To examine the TLR4 specific role of TAK242, confluent cultures of fibroblast cultures were incubated with Fn^EDA^ for 72 h in presence or absence of TAK242 to block TLR4-mediated signaling. In response to TAK242, fibroblasts showed reduced collagen and αSMA levels as shown by real-time qPCR and immunofluorescence (Figures 6A,B). Importantly, TLR4 mutant mouse (nonfunctional TLR4) skin fibroblasts showed no inhibition of Fn^EDA^-induced COL1A1-luc promoter activity with TAK242 treatment compared to wt control (Supplementary Figure 1B).

### TAK242 treatment mitigates the activated SSc fibroblast phenotype

**Figure 7:**
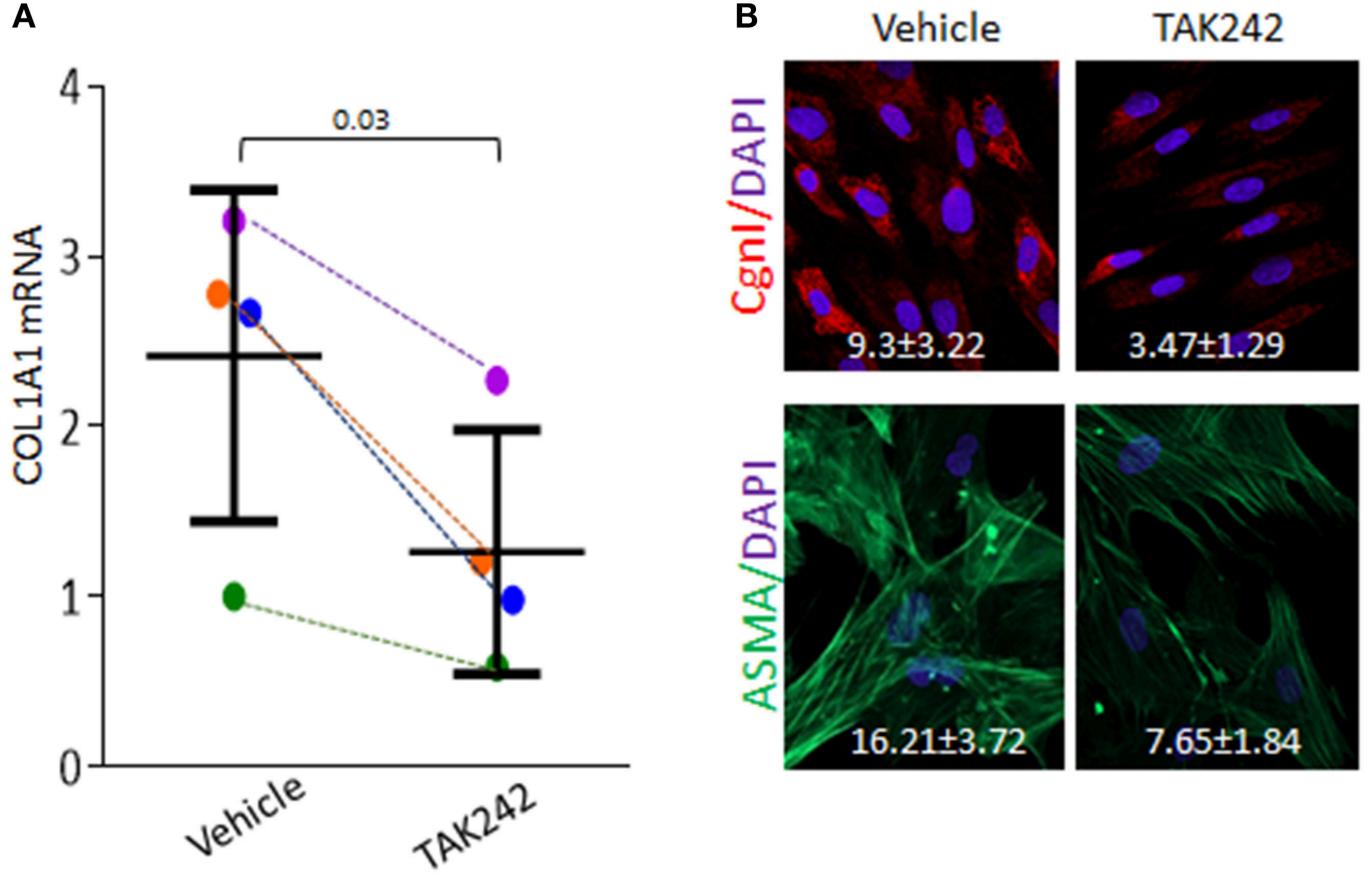
TAK242 attenuates the activated SSc fibroblast phenotype. Confluent cultures of early-passage SSc skin fibroblasts were incubated in media with TAK242 (10 μM) or vehicle for 24 h. **(A)** Real-time qPCR. Results, normalized with GAPDH, are means ± s.d. compared to vehicle-treated controls. **(B)** Immunofluorescence using antibodies to type I collagen (red) and αSMA (green). Representative images. Bar = 50 μm. Inset, quantification of immunofluorescence. Quantification of fluorescence intensities using ImageJ (means from four randomly selected hpf from four SSc fibroblasts). Paired *t*-test. Each dot and color represent an individual.

To examine the cell-autonomous role of TLR4 in the persistently activated SSc fibroblast phenotype, we established confluent cultures of fibroblast monolayers from four different SSc donors (Supplementary Table 1). Cultures were incubated with TAK242 for 24 h to block TLR4-mediated signaling. In response to TAK242, each SSc cell line showed reduced collagen gene expression (mean = 50%, *p* < 0.05) and αSMA levels (Figures 7A,B). In contrast, healthy donor showed no significant inhibition of collagen and αSMA levels (Supplementary Figure 2).

## Discussion

In contrast to more common organ-based fibrotic diseases, fibrosis in SSc synchronously affects the skin and internal organs such as lungs and heart (15, 16). Currently, there are limited therapeutic options for patients with SSc (17, 18). Immunomodulatory treatments that are highly effective in rheumatoid arthitis and other inflammatory diseases generally show only modest and variable efficacy in SSc, and at best slow disease progression (1). As dysregulated TLR4 signaling underlies the pathology of diverse chonic conditions, substantial effort has been devoted to the design and development of selective TLR4 inhibitors. TAK-242 was developed as a small-molecule selective inhibitor of TLR4 signaling that suppresses the increase in serum cytokine levels TNF-a, IL-1, and IL-6 in murine and porcine models of sepsis, as well as in LPS challenged healthy volunteers (9–11).

Disease heterogeneity is a hallmark of SSc and contributes to the lack of effective therapies to date (1). Our previous studies implicated both TLR4 and its certain endogenous “damage-associated” ligands in the pathogenesis of fibrosis in SSc (5–7). Using an experimentally-derived “fibroblast TLR4-responsive gene signature” to interrogate SSc skin biopsies, we found that biopsies displaying a strong TLR4 gene signature largely mapped to the previously defined inflammatory intrinsic gene subset of patients (7, 8, 19). Fibroblast TLR4 signatures might therefore represent biomarkers of on-going TLR4 activity; and might have potential utilities to identify SSc patients potentially responsive to TLR4 inhibition. Drug repurposing using TAK242 thus appears to be an attractive anti-fibrotic strategy for treating fibrosis in patients in SSc subset demonstrating inflammatory intrinsic gene signatures. We therefore sought to investigate the anti-fibrotic effect of TAK242 in preclinical models of organ fibrosis. Chonic subcutaneous bleomycin injection induces a local inflammatory response in mice that is followed by fibrosis in the skin and lungs. Treatment with TAK242 exerted potent antifibrotic effects in both bleomycin-induced skin fibrosis that recapitulate the inflammatory stage of SSc in fibrotic skin (20) and in the Tsk1/+ mice that resembles non-inflammatory SSc (5), and attenuated the constitutively-activated phenotype of SSc fibroblasts in culture. Moreover, the antifibrotic effects of TAK242 were not restricted to preventive application, but also when treatment was initiated after fibrosis had already been established. Most importantly, TAK242 exhibited marked efficacy in bleomycin-induced lung fibrosis, accounting for ~30% of all SSc deaths. Anti-fibrotic effects of TAK242 have been reported in experimental models of hepatic, kidney and myocardial fibrosis in preclinical treatment regimen (21–23). Our presented data demonstrate efficacy of TAK242 in complimentary inflammation-dependent and -independent preclinical models of organ fibrosis in preventive as well as in therapeutic settings.

Together, our results indicate that a novel small molecule selectively targeting TLR4 signaling prevented as well as reversed organ fibrosis in a variety of preclinical disease models, and abrogated fibrotic responses in SSc fibroblasts *in vitro*. Transcriptome-based selection of SSc patients demonstrating on-going TLR4 activity, therefore, might provide entirely new opportunities for safe and effective targeted therapy of SSc and other chonic fibrosing conditions.

## Ethics statement

Animal experiments were performed according to institutionally approved protocols and in compliance with guidelines of the Northwestern University Animal Care and Use Committee. All subjects gave written informed consent in accordance with the Declaration of Helsinki. The protocol was approved by the IRB.

## Author contributions

SB and JV conceived the project, designed experiments and interpreted. SB and JV wrote the manuscript. WW, ZT, and BS performed the major experiments, data acquisition and analysis. YT and MY contributed to reagents materials analysis tools and critical reading of the manuscript. AY performed lung histological and pathological analyses and interpretation of the results. SB and ZT performed statistical analysis.

### Conflict of interest statement

The authors declare that the research was conducted in the absence of any commercial or financial relationships that could be construed as a potential conflict of interest. The handling Editor and reviewer PG declared their involvement as co-editors in the Research Topic, and confirm the absence of any other collaboration.

## References

[1] Allanore Y Simms R Distler O Trojanowska M Pope J Denton CP. Systemic sclerosis. Nat Rev Dis Primers (2015) 1:15002. 10.1038/nrdp.2015.2 27189141

[2] Wynn TA Ramalingam TR. Mechanisms of fibrosis: therapeutic translation for fibrotic disease. Nat Med. (2012) 18:1028–40. 10.1038/nm.2807 22772564 PMC3405917

[3] Bhattacharyya S Wei J Varga J. Understanding fibrosis in systemic sclerosis: shifting paradigms, emerging opportunities. Nat Rev Rheumatol. (2011) 8:42–54. 10.1038/nrrheum.2011.149 22025123 PMC3954787

[4] Bhattacharyya S Tamaki Z Wang W Hinchcliff M Hoover P Getsios S. FibronectinEDA promotes chronic cutaneous fibrosis through Toll-like receptor signaling. Sci Transl Med. (2014) 6:232ra50. 10.1126/scitranslmed.3008264 24739758 PMC4414050

[5] Bhattacharyya S Wang W Morales-Nebreda L Feng G Wu M Zhou X. Tenascin-C drives persistence of organ fibrosis. Nat Commun. (2016) 7:11703. 10.1038/ncomms11703 27256716 PMC4895803

[6] Bhattacharyya S Kelley K Melichian DS Tamaki Z Fang F Su Y. Toll-like receptor 4 signaling augments transforming growth factor-beta responses: a novel mechanism for maintaining and amplifying fibrosis in scleroderma. Am J Pathol. (2013) 182:192–205. 10.1016/j.ajpath.2012.09.007 23141927 PMC3538029

[7] Bhattacharyya S Midwood KS Yin H Varga J. Toll-like receptor-4 signaling drives persistent fibroblast activation and prevents fibrosis resolution in scleroderma. Adv Wound Care (2017) 6:356–69. 10.1089/wound.2017.0732 29062592 PMC5649394

[8] Bhattacharyya S Varga J. Endogenous ligands of TLR4 promote unresolving tissue fibrosis: Implications for systemic sclerosis and its targeted therapy. Immunol Lett. (2018) 195:9–17. 10.1016/j.imlet.2017.09.011 28964818 PMC5820196

[9] Matsunaga N Tsuchimori N Matsumoto T Ii M. TAK-242 (resatorvid), a small-molecule inhibitor of Toll-like receptor (TLR) 4 signaling, binds selectively to TLR4 and interferes with interactions between TLR4 and its adaptor molecules. Mol Pharmacol. (2011) 79:34–41. 10.1124/mol.110.068064 20881006

[10] Takashima K Matsunaga N Yoshimatsu M Hazeki K Kaisho T Uekata M. Analysis of binding site for the novel small-molecule TLR4 signal transduction inhibitor TAK-242 and its therapeutic effect on mouse sepsis model. Br J Pharmacol. (2009) 157:1250–62. 10.1111/j.1476-5381.2009.00297.x 19563534 PMC2743844

[11] Goldfarb RD Ortegel JW Parrillo JE Zanotti-Cavazzoni S Casey LC Dellinger RP. TAKEDA-143242 increased survival via reduced cytokines in porcine peritonitis. J Surg Res. (2011) 166:e165–73. 10.1016/j.jss.2010.09.031 21236445

[12] Rice TW Wheeler AP Bernard GR Vincent JL Angus DC Aikawa N. A randomized, double-blind, placebo-controlled trial of TAK-242 for the treatment of severe sepsis. Crit Care Med. (2010) 38:1685–94. 10.1097/CCM.0b013e3181e7c5c9 20562702

[13] Hubner RH Gitter W El Mokhtari NE Mathiak M Both M Bolte H. Standardized quantification of pulmonary fibrosis in histological samples. BioTechniques (2008) 44:507–11; 514–7. 10.2144/000112729 18476815

[14] Sakai N Nakamura M Lipson KE Miyake T Kamikawa Y Sagara A. Inhibition of CTGF ameliorates peritoneal fibrosis through suppression of fibroblast and myofibroblast accumulation and angiogenesis. Sci Rep. (2017) 7:5392. 10.1038/s41598-017-05624-2 28710437 PMC5511333

[15] Gabrielli A Avvedimento EV Krieg T. Scleroderma. N Engl J Med. (2009) 360:1989–2003. 10.1056/NEJMra0806188 19420368

[16] Ho YY Lagares D Tager AM Kapoor M. Fibrosis–a lethal component of systemic sclerosis. Nat Rev Rheumatol. (2014) 10:390–402. 10.1038/nrrheum.2014.53 24752182

[17] Allanore Y Matucci-Cerinic M Distler O. Treatment of systemic sclerosis: is there any hope for the future? RMD Open. (2016) 2:e000260. 10.1136/rmdopen-2016-000260 27486527 PMC4947768

[18] Denton CP Khanna D. Systemic sclerosis. Lancet (2017) 390:1685–99. 10.1016/S0140-6736(17)30933-9 28413064

[19] Hinchcliff M Huang CC Wood TA Matthew Mahoney J Martyanov V Bhattacharyya S. Molecular signatures in skin associated with clinical improvement during mycophenolate treatment in systemic sclerosis. J Investig Dermatol. (2013) 133:1979–89. 10.1038/jid.2013.130 23677167 PMC3714324

[20] Sargent JL Li Z Aliprantis AO Greenblatt M Lemaire R Wu MH. Identification of optimal mouse models of systemic sclerosis by interspecies comparative genomics. Arthritis Rheumatol. (2016) 68:2003–15. 10.1002/art.39658 26945694 PMC5695038

[21] Wen Z Ji X Tang J Lin G Xiao L Liang C. Positive feedback regulation between transglutaminase 2 and toll-like receptor 4 signaling in hepatic stellate cells correlates with liver fibrosis post *Schistosoma japonicum* Infection. Front Immunol. (2017) 8:1808. 10.3389/fimmu.2017.01808 29321784 PMC5733538

[22] Chen L Sha ML Li D Zhu YP Wang XJ Jiang CY. Relaxin abrogates renal interstitial fibrosis by regulating macrophage polarization via inhibition of Toll-like receptor 4 signaling. Oncotarget (2017) 8:21044–53. 10.18632/oncotarget.15483 28416741 PMC5400564

[23] Zhang Y Peng W Ao X Dai H Yuan L Huang X. TAK-242, a toll-like receptor 4 antagonist, protects against aldosterone-induced cardiac and renal injury. PLoS ONE (2015) 10:e0142456. 10.1371/journal.pone.0142456 26556241 PMC4640881

