# Supplementary material for: Pharmacological Inhibition of Toll-Like Receptor-4 Signaling by TAK242 Prevents and Induces Regression of Experimental Organ Fibrosis
Source: Front Immunol. 2018 Oct 23;9:2434. doi: 10.3389/fimmu.2018.02434 (PMC6207051; doi:10.3389/fimmu.2018.02434)

Supplementary Figure 1.

Normal skin fibroblasts were incubated with TAK242 (as indicated) for indicated periods. **A.** After 24 h, cytotoxicity was determined (percent of LDH release). **B.** Fibroblasts from wt and TLR4 mutant mice skin transiently transfected with COL1A1-luc were incubated in medium with endotoxin-stripped Fn^{EDA} (1 ug/ml) in the presence or absence of TAK242 (3uM) for 24 h. Whole-cell lysates were assayed for their luciferase activities. Results are means ± SD from thee determinations. *$P = 0.004$, Fn^{EDA} alone versus treated with TAK242 in wt. P =ns, Fn^{EDA} alone versus treated with TAK242 in TLR4 mutant. Sidak's multiple comparison test. ns, not significant.

Supplementary Figure 2.

Fibroblasts from three healthy donors' skin were incubated in medium with TAK242 (3uM) for 24 h and mRNA levels were determined by real-time qPCR. Results, normalized with GAPDH, are means ± s.d. compared to vehicle-treated controls. Control versus TAK242; Paired t-test. P = ns. not significant. Each dot and color represents an individual.

Supplementary Table 1

SSc fibroflasts derived from the subjects indicated below

Sample ID	Age	Sex	dc/lc	Disease stage	MRSS
SSc1107	48	M	dcSSc	Early	25
SSc1121	47	F	dcSSc	Late	26
SSc1068	49	F	dcSSc	Late	27
SSc1097	64	F	dcSSc	Early	9

dcSSc diffuse cutaneous SSc; F, female; M, male; mRSS, modified Rodnan skin score (1 to 51).

Supplementary Fig. 1

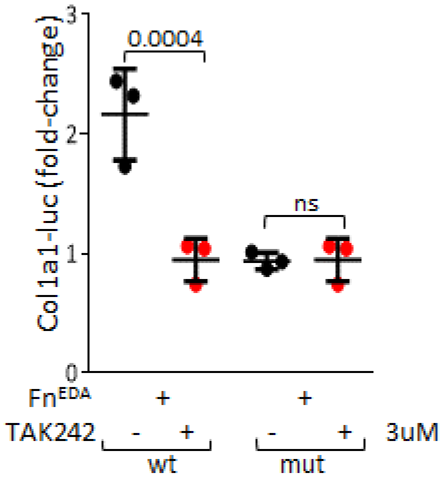

Supplementary Fig. 2

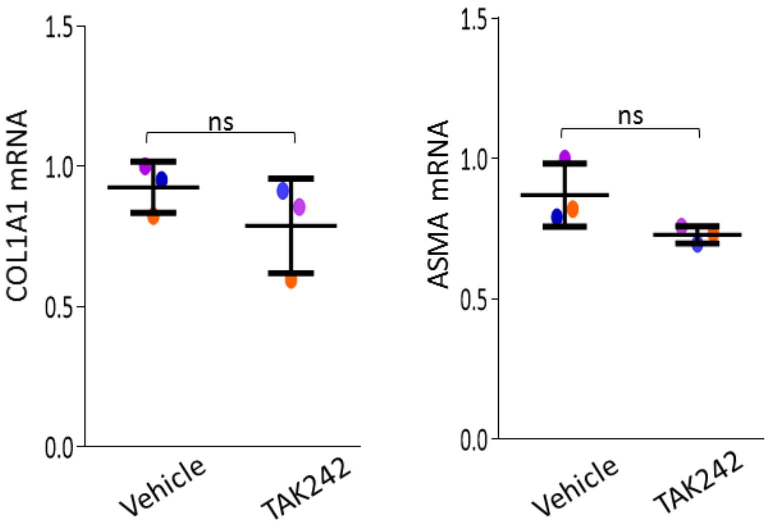

Supplement: Supplementary file 1 [file Table_1.pdf]
